# Small Media and Client Reminders for Colorectal Cancer Screening: Current Use and Gap Areas in CDC’s Colorectal Cancer Control Program

**DOI:** 10.5888/pcd9.110317

**Published:** 2012-07-19

**Authors:** Matthew W. Kreuter, Lori B. Garibay, Debbie J. Pfeiffer, Jennifer C. Morgan, Melonie Thomas, Katherine M. Wilson, Jennifer Pieters, Kellie Szczepaniec, Amy Scott, Timothy J. Poor

**Affiliations:** Author Affiliations: Lori B. Garibay, Debbie J. Pfeiffer, Jennifer C. Morgan, Kellie Szczepaniec, Amy Scott, Timothy J. Poor, Washington University in Saint Louis, Saint Louis, Missouri. Melonie Thomas, Katherine M. Wilson, Jennifer Pieters, Centers for Disease Control and Prevention, Atlanta, Georgia.

## Abstract

**Introduction:**

CDC’s Colorectal Cancer Control Program (CRCCP) funds 25 states and 4 tribal organizations to promote and increase colorectal cancer screening population-wide. The CRCCP grantees must use evidence-based strategies from the *Guide to Community Preventive Services*, including small media and client reminders.

**Methods:**

To assess the existing resources and needs to promote colorectal cancer screening, we conducted 2 web-based surveys of CRCCP grantees and their community partners. Survey 1 sought to identify priority populations, the number and quality of existing colorectal cancer resources for different population subgroups, and the types of small media and client reminder they were most interested in using. Survey 2 assessed screening messages that were used in the past or might be used in the future, needs for non-English–language information, and preferences for screening-related terminology.

**Results:**

In survey 1 (n = 125 from 26 CRCCPs), most respondents (83%) indicated they currently had some information resources for promoting screening but were widely dissatisfied with the quality and number of these resources. They reported the greatest need for resources targeting rural populations (62% of respondents), men (53%), and Hispanics (45%). In survey 2 (n = 57 from 25 CRCCPs), respondents indicated they were most likely to promote colorectal cancer screening using messages that emphasized family (95%), role models (85%), or busy lives (83%), and least likely to use messages based on faith (26%), embarrassment (25%), or fear (22%). Nearly all (85%) indicated a need for resources in languages other than English; 16 different languages were mentioned, most commonly Spanish.

**Conclusion:**

These findings provide the first picture of CRCCP information resources and interests, and point to specific gaps that must be addressed to help increase screening.

## Introduction

The Centers for Disease Control and Prevention’s (CDC’s) Colorectal Cancer Control Program (CRCCP) (http://www.cdc.gov/cancer/crccp) funds 25 states and 4 tribal organizations to increase population screening rates to 80% by 2014 (1). To achieve this goal, CRCCPs are encouraged to promote screening using evidence-based approaches such as small media and client reminders from the *Guide to Community Preventive Services* (1,2). The purpose of this study is to assess CRCCP’s current use of and needs and priorities for these evidence-based approaches.

*Small media* such as patient education materials inform and educate people about cancer screening and motivate them to seek information, talk to a doctor, make an appointment and get screened. Small media can be mailed, e-mailed or distributed through waiting rooms or examination rooms, where they are picked up and taken away by patients or clients. Small media are especially effective when targeted to a specific audience (3) and are recommended by the Task Force on Community Preventive Services based on “strong evidence” of increasing mammography, Papanicolaou (Pap) testing, and fecal occult blood testing (FOBT) (4,5).

*Client reminders* are letters or postcards that inform or remind patients and clients about a needed screening. They can target clients newly eligible for screening (ie, turning 50), due or overdue for screening, or who need to make or keep an appointment for screening. Client reminders can be delivered by mail, telephone, e-mail or text message, and are especially useful when contact lists are available for large groups of screening-eligible clients, and when reminders can be sent by a source known to and trusted by the recipient. The Task Force recommends client reminders based on “strong evidence” for mammography and “sufficient evidence” for Pap testing and FOBT (6).

## Methods

We administered 2 web-based surveys to the 25 state agencies and 4 tribal organizations that were awarded CRCCP cooperative agreements from CDC. Survey 1 sought to identify priority populations, the number and quality of existing colorectal cancer resources for different population subgroups, and the types of small media and client reminder they were most interested in using. Survey 2 assessed screening messages they had used in the past or were interested in using now, needs for non-English–language information, and preferences for screening-related terminology. The Washington University Human Research Protection Office reviewed the study protocol and judged the research to be exempt.

To inform development of the surveys, we conducted an environmental scan of publicly available information resources promoting colorectal cancer (CRC) screening. The goals were to identify types of small media and client reminders currently in use (survey 1) and message themes, taglines, and calls to action used in these resources (survey 2). We conducted an online search in March and April 2010. Search terms combined information about place (eg, name of state or tribe) with topic (eg, CRC, colon, colorectal cancer, screening, testing) and resource type (eg, tools, materials). For each state, we also searched “comprehensive cancer control” and for tribal organizations, we also searched by racial category (eg, American Indian/Alaska Native), including derivatives and synonyms (eg, Native American, indigenous people). We identified and reviewed 235 resources from 36 states, federal agencies, and tribal and other organizations. The types of resources, message themes, and calls to action identified in this review helped shape items and response options in the surveys.

CDC has awarded CRCCP cooperative agreements to 25 states and 4 tribal organizations. Three of these received awards after survey 1 was completed and thus are not included in that sample. The sampling frame for survey 1 included the program director, coordinator and core staff from each CRCCP (“CRCCP leaders”) as well as community partners they identified as key collaborators on CRC screening, outreach, awareness, education, or recruitment. This latter group included community health centers, local health departments, health care providers and networks, and cancer coalitions and advocacy organizations. Links to the online survey were sent to 84 CRCCP leaders and 204 community partners. The sampling frame for survey 2 included only CRCCP leaders (n = 98).

The anonymous surveys were administered using Qualtrics survey software (Qualtrics Laboratories Inc, Provo, Utah). CRCCP leaders were invited to participate by e-mail and accessed the survey via an embedded web link. They received a separate web link to share with their community partners. Survey 1 was administered from April to June 2010. It included 38 questions, of which respondents could answer from 9 to 38, depending on skip patterns. Most respondents (90%) completed the survey within 20 minutes. Survey 2 was administered from October to November 2010, included 49 to 56 questions, and on average took 34 minutes to complete.

In both surveys, resources were defined as information used to promote colorectal cancer awareness and screening, including print materials, messages or media spots. Survey items were developed specifically for this study.

### Survey 1

Respondents were first asked if their organization had any resources promoting CRC screening (yes, no, not sure). Those responding yes were asked which of 7 audience types their resources were intended to reach: patients, health care providers, community health centers, community organizations, and wives/partners, husbands/partners, or children of screening-eligible adults). For each audience type selected, they were asked how many different resources they had (1, 2, 3 or more) and how satisfied or dissatisfied they were with the number and quality of these resources (5-point scale: 1 = very dissatisfied; 2 = dissatisfied; 3 = neither satisfied nor dissatisfied; 4 = satisfied; 5 = very satisfied). Finally, for each audience type, they were asked whether the resources they had were further targeted to racial/ethnic groups (African American, Caucasian, Asian/Native Hawaiian/Pacific Islander, American Indian/Alaska Native, Hispanic/Latino), men or women, place of residence (urban/rural) or low-income populations (subjectively defined by the respondent).

Respondents were asked to think about their organization’s needs for targeted colorectal cancer resources over the next 2 years and rank their top 3 priority populations from the following categories: urban, rural, men, women, Caucasian, African American, Hispanic/Latino, Asian/Pacific Islander/Native Hawaiian, American Indian/Alaska Native, or other.

Based on the environmental scan, we identified 19 types of small media and 7 types of client reminders for promoting colorectal cancer screening. Respondents selected up to 3 from each list that their organization would be most interested in using. Small media were inserts, business cards, lottery-style scratch off cards, church fans, brochures, newsletters, tip sheets, patient education materials, letters, flyers, posters, text messages, checklists, booklets, bookmarks, question lists for provider visits, frequently asked questions (FAQs), self-administered quizzes, and other types of client reminders. Client reminders were greeting cards, postcards, letters, telephone message scripts, radio public service announcement (PSA) scripts, videos, and other.

Respondents checked up to 3 types of content their organization was most interested in using (screening options, procedure preparation, procedure aftercare, coverage under health care reform, screening criteria, risk factors, prevention behavior, and other).

### Survey 2

Respondents viewed 8 sample resources promoting colorectal cancer (CRC) screening, each using a different message theme identified in the environmental scan (family, fear, role models, lighthearted or humor, embarrassment, faith-based, busy lives, and 50th birthday). Although in practice some of these themes might be combined (eg, a role model message for people with busy lives), our survey treated them as distinct for simplicity of measurement. After seeing each example, respondents were asked if their organization had ever used this theme in health information materials about any topic (yes, no, not sure) and about CRC screening specifically (yes, no, not sure). Next they viewed message concepts about CRC screening that used each theme. For example, “If you wait for symptoms, it may be too late” (a concept based on the theme fear) and “Cancer doesn’t care that you’re busy” (a concept based on the theme busy lives). The number of message concepts ranged from 6 to 13 across themes, and respondents rated the likelihood of using each (very likely to use it, not likely to use it, no strong opinion). Using the same response scale, respondents rated 8 taglines, or calls to action, such as “If you’re 50 or older, you should be tested for colon cancer” and “Talk to your doctor about scheduling an appointment.”

In separate questions, respondents indicated which term their organization preferred to use for FOBT and colonoscopy (*screening*, *testing*, no preference), and cancer type (*colorectal, colon,* no preference). Respondents indicated whether their organization needed CRC materials in languages other than English (yes, no, not sure) and if so, specified the language(s) needed. We used descriptive statistics to characterize survey responses. Differences between the responses of CRCCP leaders and community partners were compared using χ^2^ and *t*-tests. On both surveys, data were missing for some respondents who did not complete the entire survey (20% for survey 1, 29% for survey 2). In descriptive analyses, we included all data available for each item. In bivariate and comparative analyses, we included only respondents who answered all questions involved in the analysis.

## Results

### Survey 1

Responses were received from 69 of 84 CRCCP leaders (82%) and 56 of 204 community partners (28%). Most (66%) had been in their current position more than 2 years. Community partners were most commonly from community health centers (45%), local or nonprofit agencies (36%), and other community organizations (18%). Respondents represented 25 of 26 CRCCPs (96%).

Most respondents (83%) reported having at least some resources promoting CRC screening, although it was more common among CRCCP leaders than their community partners (90% vs 74%; *P* = .015). Most commonly, these resources were intended to reach patients (95%), health care providers (70%), wives (48%), husbands (48%), community health centers (47%), or other community organizations (46%). Satisfaction with the number and quality of these resources was low. On a 5-point scale (1 = very dissatisfied to 5 = very satisfied), mean responses ranged from 2.5 to 2.7 for *number* of resources and 2.2 to 2.4 for *quality* of resources (Table).

**Table:** Current Colorectal Cancer Screening Resources Reported by Survey Respondents, Centers for Disease Control and Prevention’s Colorectal Cancer Control Program, 2010

Survey Questions	Resources Designed for . . . ^a^			

	Patients	Health Care Providers	Community Health Centers	Community Organizations
Have any resources? % yes (n = 99)	94.9	69.7	46.5	45.5
**Number of different resources, %**				
1	23.7	58.1	55.9	61.1
2	21.5	16.1	26.5	13.9
≥3	54.8	25.8	17.6	25.0
**Satisfaction^b^**				
With *number* of resources	2.5	2.6	2.7	2.5
With *quality* of resources	2.2	2.4	2.3	2.4
**Resources targeted to . . . , % yes^a^**				
**Race/ethnicity**				
Caucasian	32.0	24.6	23.9	33.3
African American	36.2	23.2	30.4	33.3
Asian/Pacific Islander/Native Hawaiian	5.3	8.7	8.7	11.1
American Indian/Alaska Native	19.1	17.4	19.6	22.2
Hispanic/Latino	43.6	29.9	37.5	35.6
**Sex**				
Male	51.1	36.2	41.3	46.7
Female	50.0	36.2	41.3	46.7
**Residence**				
Urban	23.4	23.2	26.1	27.6
Rural	27.7	21.7	23.1	31.1
Low-income	40.4	33.3	43.5	46.7

^a^ Denominator includes only those reporting yes to “Have any resources?”

^b^ Satisfaction scored on a 5-point scale: 1 = very dissatisfied; 2 = dissatisfied; 3 = neither satisfied nor dissatisfied; 4 = satisfied; 5 = very satisfied.

**Figure 1:**
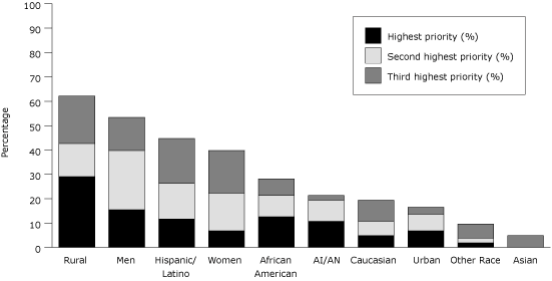
Priority Needs for Information Resources About Colorectal Cancer Screening, by Population

Respondents identified rural populations (62%), men (53%), Hispanics (45%), and women (40%) as populations for which they most needed targeted resources (Figure 1).

**Table:** 

Population	Highest Priority, %	Next Highest Priority, %	Third Highest Priority, %	Cumulative Percentage
**Rural**	29.1	13.6	19.4	62.1
**Men**	15.5	24.3	13.6	53.4
**Hispanic/Latino**	11.7	14.6	18.4	44.7
**Women**	6.8	15.5	17.5	39.8
**African American**	12.6	8.7	6.8	28.2
**AI/AN**	10.7	8.7	1.9	21.4
**White**	4.9	5.8	8.7	19.4
**Urban**	6.8	6.8	2.9	16.5
**Other**	1.9	1.9	5.8	9.7
**Asian**	0	0	4.9	4.9

For small media, respondents (n = 102) were most interested in using patient education materials (46% of respondents), brochures (26%), posters (23%), questions to ask providers (19%), and inserts (18%). Few participants were interested in using newsletters (3%), text messages (3%), or bookmarks (2%). For client reminders, respondents (n = 98) were most interested in postcards (56%), radio PSA scripts (35%), letters (29%), greeting cards (28%), and telephone message reminder scripts (21%).

Respondents were most interested in information about prevention behavior (52%), screening options (45%), risk factors (43%), screening criteria (28%), coverage under health care reform (26%), and procedure preparation (24%).

### Survey 2

Responses were received from 57 of 98 CRCCP leaders (58%) representing 25 of 29 CRCCPs (86%). More than half (55%) had been in their position for more than 2 years.

Most respondents reported that their organization had previously used busy lives (61%), family-based (60%), and role model (52%) message themes to promote CRC screening. Fewer respondents reported having used message themes based on humor or lightheartedness (39%), 50th birthdays (27%), fear (18%), faith (14%) or embarrassment (6%).

**Figure 2:**
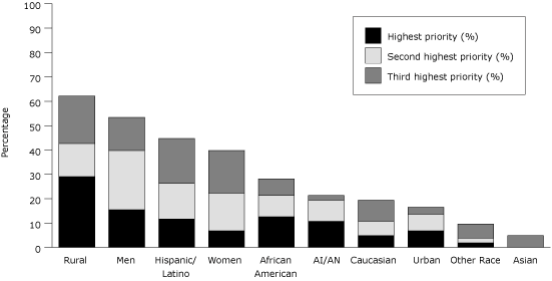
Likelihood of Using 8 Message Themes to Promote Colorectal Cancer Screening

When respondents viewed message concepts from each of these themes, the proportion classified as “very likely to use” was highest for the family and embarrassment themes (both 42%), followed by role models (35%), busy lives (34%), 50th birthdays (33%), humor or lightheartedness (27%), fear (26%), and faith (18%). After rating 6 to 13 message concepts about each message theme, respondents indicated how likely their organization was to use each theme in future screening materials. Responses varied by theme (Figure 2); respondents were most likely to use “family” and least likely to use “fear” as message themes.

**Table:** 

Message Theme	% Very Likely to Use	% Likely to Use	Cumulative %^a^
**Family (n = 55)**	58	36	95
**Role model (n = 54)**	31	54	85
**Busy lives (n = 53)**	30	53	83
**50th birthday (n = 53)**	19	60	79
**Lighthearted (n = 53)**	17	36	53
**Faith (n = 53)**	6	21	26
**Embarrassment (n = 53)**	4	21	25
**Fear (n = 55)**	5	16	22

^a^ May not equal sum of columns because of rounding decimals.

Nearly all respondents indicated that they would be likely to use the following taglines: “If you’re 50 or older, you should be tested for colon cancer” (90%) and “Talk to your doctor and get screened” (87%). Fewer respondents endorsed taglines that were less directive and explicit about screening: “Talk to your doctor to learn more” (64%) and “Talk to your doctor about scheduling an appointment” (54%).

Most respondents reported that their organization used the term *colorectal* (54%) vs *colon* (27%) cancer; 19% indicated no strong preference. For colonoscopy, there was a strong preference for the term *screening* (75%) vs *testing* (19%). For FOBT, 58% preferred *screening*, 21% preferred *testing,* and 21% had no preference.

Nearly all respondents (85%) indicated needing materials in languages other than English. Most specified a need for Spanish language resources (65%), followed by Creole (9%), Vietnamese, Korean, and Arabic (5% each). Sixteen different languages were specified.

## Discussion

This study identifies at least 3 ways to enhance the efforts of CRCCP leaders and their community partners in using small media and client reminders to promote CRC screening.

First, they need more and better information resources for the populations they serve. Although most CRCCP leaders reported having at least some resources to promote CRC screening, fewer of their community partners did. Resources were mostly designed for patients; one-third of respondents had no resources for providers and half had no resources for community health centers. Furthermore, satisfaction with the number and quality of these resources was low — mean ratings were closer to *dissatisfied* than *satisfied*.

Second, CRCCPs need resources targeted to diverse population subgroups. Few of their existing resources are designed for racial, ethnic, rural, and low-income populations. Resources targeted to men, rural populations, and Hispanics were needed most, as were Spanish-language materials. These gaps correspond closely to disparities in CRC screening; lower rates are found among racial/ethnic minorities (especially Hispanics), low-income populations, and some rural populations (7-10).

Third, CRCCPs might expand their arsenal of strategies, using more and different types of evidence-based interventions and message strategies to promote screening. For example, respondents indicated little interest in strategies such as text messaging or message concepts like faith or fear, even though some empirical evidence suggests that these approaches change cancer-related behaviors, including screening (11-13). This may be because technical knowledge or capacity is lacking (eg, for texting), because certain message strategies have not tested well in agencies’ audience research (eg, fear messages) or because grantees are more comfortable using approaches they have used in the past. But study findings also suggest that CRCCP leaders are willing to try new things. In survey 2 many reported that their organizations had not used certain message strategies in the past, yet after viewing samples of these strategies, they expressed interest in using them. This finding suggests a desire by many public health departments to evolve and be forward thinking, and reflects an opportunity to increase evidence-based practices by better sharing promising ideas with practitioners.

The findings underscore the variability across CRCCPs. While all share a common mission, each serves a different mix of population subgroups with different language needs, using different strategies and messages, even different CRC terminology. National programs like CRCCP must balance the desire for a common campaign identity with the need to help state and local agencies customize resources to best serve their local mix of constituents. For example, CRCCP leaders and their partners must choose specific types of small media and client reminders and adapt them for different settings and populations. In business, this challenge is met using “mass customization” or “co-creation” systems, in which consumers design their own version of standard products like shoes or tote bags (14,15). Similar approaches have helped community organizations deliver cancer information (16) and might be a fit for CRCCPs.

The inclusion of community partners in survey 1 reflects their important role promoting and delivering screening. They have direct contact with patients and populations and can use or ignore evidence-based approaches just as state and tribal organizations can. Their response rates were markedly lower than those for CRCCP leaders (28% vs 82%). It is possible they felt less obligated to complete the survey than did state and tribal organizations with direct fiscal and programmatic ties to CRCCP, although we collected no data to support or refute this explanation.

Although we make no claims about the generalizability of findings beyond CRCCPs and their community partners, we suspect the findings are not unique to this sample. Our environmental scan — which identified CRC information resources from a much broader set of organizations — revealed similar findings: the quality of many resources was quite low and very few materials were targeted to specific population subgroups. Any efforts by CDC or other leading cancer control agencies to address the information needs identified in these surveys could benefit all CRC-related organizations, not just CRCCPs. Even if a broader benefit is not realized, the study’s focus on a new national cancer control program carried out by a large group of state and local organizations is justified. Understanding how such programs work and how they can be improved will enhance their effectiveness and help guide development of future large-scale public health efforts.

The surveys gathered limited respondent data, which precluded exploring some of the ways that responses might have varied by characteristics of the sample or their organizations. For example, although we found some differences between the responses of CRCCP leaders and their community partners (eg, in availability of resources to promote CRC), organizations might also vary in the extent to which CRC screening was central to their missions (compared with a broader focus on all cancers or health in general) or in their level of past experience working to promote cancer screening. Nor did the survey examine how CRCCPs planned to integrate these small media and client reminder strategies into broader efforts to increase CRC screening population-wide. Coordinating communication activities with policy and system changes should make all of them more effective and is part of the strategic approach for CRCCPs (3).

Findings from these surveys help in understanding and prioritizing the needs of public health agencies and their community partners in implementing evidence-based approaches to increase CRC screening. These needs might best be met by a multilevel approach that includes developing systems to help practice agencies promote long-lasting protective interventions (ie, screening), using effective and audience-specific information and education (17). There are clear gaps to fill in assuring that high-quality, audience-appropriate information resources that use proven approaches are available for all Americans. Developing appropriate systems and supports to fill these gaps should be a priority in helping meet the nation’s goals for colorectal cancer control.

## References

[1] Department of Health and Human Services, Centers for Disease Control and Prevention, Integrating colorectal cancer screening within chronic disease programs (CDC-RFA-DP09-903). 2009. http://www07.grants.gov/search/search.do?oppId=45233&flag2006=false&mode=VIEW. Accessed April 20, 2012.

[2] Joseph DA , DeGroff AS , Hayes NS , Wong PL , Plescia M . The Colorectal Cancer Control Program: partnering to increase popualtion level screening. Gastrointest Endosc 2011;73(3):429-34. 10.1016/j.gie.2010.12.027 21353839

[3] Guide to Community Preventive Services. Cancer prevention and control, client-oriented screening interventions: small media; 2009. http://www.thecommunityguide.org/cancer/screening/client-oriented/smallmedia.html. Accessed April 20, 2012.

[4] Baron RC , Rimer BK , Coates RJ , Kerner J , Kalra GP , Melillo S , Client-directed interventions to increase community access to breast, cervical, and colorectal cancer screening: a systematic review. Am J Prev Med 2008;35(1):S56-66. 10.1016/j.amepre.2008.04.001 18541188

[5] Task Force on Community Preventive Services. Recommendations for client- and provider-directed interventions to increase breast, cervical, and colorectal cancer screening. Am J Prev Med 2008;35(1s)S21-5. 10.1016/j.amepre.2008.04.004 18541184

[6] Guide to Community Preventive Services. Cancer prevention and control, client-oriented screening interventions: client reminders. http://www.thecommunityguide.org/cancer/screening/client-oriented/reminders.html. Accessed April 20, 2012.

[7] Jerant AF , Fenton JJ , Franks P . Determinants of racial/ethnic colorectal cancer screening disparities. Arch Intern Med 2008;168(12):1317-24. 10.1001/archinte.168.12.1317 18574089

[8] Meissner HI , Breen N , Klabunde CN , Vernon SW . Patterns of colorectal cancer screening uptake among men and women in the United States. Cancer Epidemiol Biomarkers Prev 2006;15(2):389-94. 10.1158/1055-9965.EPI-05-0678 16492934

[9] Vital signs: colorectal cancer screening among adults aged 50-75 years, United States, 2008. MMWR Morb Mortal Wkly Rep 2010;59(26):1-5. 20613704

[10] Fisher JL , Engelhardt HL , Stephens JA , Smith BR , Haydu GG , Indian RW , Cancer-related disparities among residents of Appalachia Ohio. J Health Dispar Res Pract 2008;2(2):61-74.

[11] Kreuter MW , Sugg-Skinner C , Holt CL , Clark EM , Haire-Joshu D , Fu Q , Cultural tailoring for mammography and fruit and vegetable intake among low-income African-American women in urban public health centers. Prev Med 2005;41(1):53-62. 10.1016/j.ypmed.2004.10.013 15916993

[12] Witte K , Allen M . A meta-analysis of fear appeals: implications for effective public health campaigns. Health Educ Behav 2000;27(5):591-615. 10.1177/109019810002700506 11009129

[13] Fjeldsoe BS , Marshall AL , Miller YD . Behavior change interventions delivered by mobile telephone short-message service. Am J Prev Med 2009;36(2):165-73. 10.1016/j.amepre.2008.09.040 19135907

[14] Pine J . Mass customization: The new frontier in business competition. Boston (MA): Harvard Business School Press; 1993.

[15] Peppers D , Rogers M . Enterprise one to one: tools for competing in the interactive age. 1st edition. New York: Currency Doubleday; 1997.

[16] Kreuter MW , Fernandez M , Richert M , Cofta-Woerpel L , Pfeiffer D , Adams-Piphus B , Increasing information-seeking about HPV vaccination through community partnerships in African American women. Fam Community Health 2011;35(1):1-16. 10.1097/FCH.0b013e3182385d13 PMC3864558 22143485

[17] Frieden TR . A framework for public health action: the health impact pyramid. Am J Public Health 2010;100(4):590-5. 10.2105/AJPH.2009.185652 20167880 PMC2836340

